# Influence of the Fibroblastic Reticular Network on Cell-Cell Interactions in Lymphoid Organs

**DOI:** 10.1371/journal.pcbi.1002436

**Published:** 2012-03-22

**Authors:** Frederik Graw, Roland R. Regoes

**Affiliations:** Institute of Integrative Biology, ETH Zurich, Zurich, Switzerland; Utrecht University, Netherlands

## Abstract

Secondary lymphoid organs (SLO), such as lymph nodes and the spleen, display a complex micro-architecture. In the T cell zone the micro-architecture is provided by a network of fibroblastic reticular cells (FRC) and their filaments. The FRC network is thought to enhance the interaction between immune cells and their cognate antigen. However, the effect of the FRC network on cell interaction cannot be quantified to date because of limitations in immunological methodology. We use computational models to study the influence of different densities of FRC networks on the probability that two cells meet. We developed a 3D cellular automaton model to simulate cell movements and interactions along the FRC network inside lymphatic tissue. We show that the FRC network density has only a small effect on the probability of a cell to come into contact with a static or motile target. However, damage caused by a disruption of the FRC network is greatest at FRC densities corresponding to densities observed in the spleen of naïve mice. Our analysis suggests that the FRC network as a guiding structure for moving T cells has only a minor effect on the probability to find a corresponding dendritic cell. We propose alternative hypotheses by which the FRC network might influence the functionality of immune responses in a more significant way.

## Author Summary

The interaction between lymphocytes and antigen presenting cells or infected cells is thought to be enhanced by the complex microarchitecture of secondary lymphoid organs such as the spleen or lymph nodes. In the T cell zone the micro-architecture is provided by a network of fibroblastic reticular cells (FRC) and their filaments which are assumed to work as a “road system” on which T cells can migrate. However, the effect of the FRC network on cell interaction cannot be quantified experimentally to date because of limitations in immunological methodology. We use computational models to study the influence of different kinds of FRC networks on the probability that two cells meet. We can show that the structure of the FRC network has only a small influence on this probability. However, disruption of the FRC network as observed in persistent infections maximally impairs the contact probability between cells in FRC densities corresponding to those observed in the spleen of naïve mice. Our analysis suggests that the FRC-network as a guiding structure has only a limited effect on the probability of a single cell to find its appropriate counterpart. Further analysis is suggested to reveal the importance of the FRC-network.

## Introduction

Secondary lymphoid organs (SLOs), such as lymph nodes (LN) or the spleen, are anatomical structures important for the establishment and proper functioning of immune responses. In the absence of these SLO, an organism fails to control an infection [1]. SLOs are strongly connected to the blood, and thus facilitate cell-cell interactions across the entire body. Of particular importance for immune responses are interactions between naïve T cells and antigen-presenting cells, such as dendritic cells (DC) [2], as well as interactions between activated T cells and infected cells.

Lymph nodes and the spleen have themselves a highly organized architecture with different anatomical compartments for specific subsets of lymphocytes (reviewed in [3], [4]). Recently developed two-photon microscopy methods enable us to observe how the cells move inside of LNs and the spleen *ex vivo* or *in vivo* [5]–[10]. Using this method, it has been observed that lymphocytes move along the fibroblastic reticular cell (FRC) network – a network formed by FRC and filaments between them [11]. Observations made by electron microscopy had already revealed the detailed structure of the FRC network which forms “corridors”, through which lymphocytes migrate [12]–[14]. The FRC network provides guidance for T cells, which preferentially move along the network filaments due to certain chemokines expressed by the FRC network [11], [14], [15]. Especially the lymphoid chemokines CCL19 and CCL21, which interact with the CCR7 receptor on naïve T cells and activated dendritic cells, and other soluble factors presented by FRCs influence T cell motility in lymph nodes [16]–[18]. Additionally, dendritic cells tend to reside on FRCs [19], [20].

It is found that the FRC network is disrupted or changed in several infections. For example, infections with the lymphocytic choriomeningitis virus (LCMV) Clone-13 strain or with visceral leishmaniasis in mice are associated with a disrupted FRC network [21], [22]. On the other hand, chronic human immunodeficiency virus (HIV) infection leads to additional deposition of collagen in the lymphoid tissue (fibrosis), consolidating the existing FRC network [23]. However, it is not clear to what extent changes in the FRC network affect the efficacy of the immune response [24], [25].

In this study, we examine quantitatively how the FRC network influences cell-cell interactions. To this end, we extended a recently published 3D cellular automaton model for lymphatic tissue to allow for a more detailed description of the FRC network [26]. We simulate the spatial movement of T cells in lymphnodes that are structured by FRC network with different characteristics. To investigate the effect of FRC networks we manipulate the density of FRCs and the number of filaments between them. We then study with what probability a T cell encounters a static dendritic cell with dynamic dendrites or a motile target. Our analysis reveals that the influence of the FRC network structure on this probability is only minor.

Based on our analysis, we conclude that enhancement of the contact probability of a single T cell with a target is not the most important contribution of the FRC network to secondary lymphoid organs to make them an efficient environment for the establishment and proper functioning of immune responses. We propose other hypotheses and experimental methods to test them in order to reveal the importance of the FRC network.

## Results

**Figure 1:**
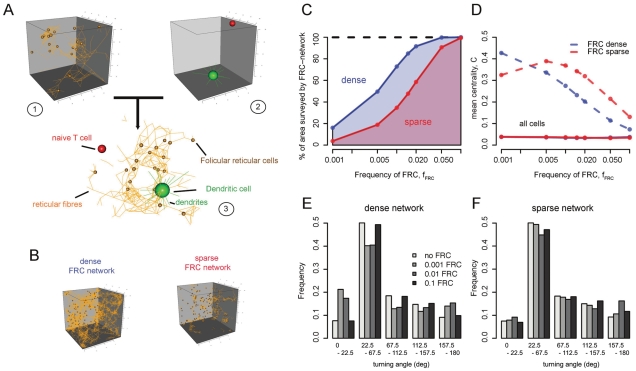
The simulation tool and the analysis of FRC network structure and motility. **A** Simulation of the FRC network (1) and cell movement (2) in two separate but interacting cellular automata (3). The sketch shows the 3D cube with a size of 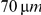 ( = 10 nodes) in each dimension. **B** A sketch of a dense and sparse FRC network in a cubic space of 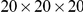 cells is shown. Plot **C** and **D** show the analysis of the FRC network structure: In **C**, we show the average fraction of the simulated area, which is surveyed by a cell crawling along each filament of the FRC network dependent on the frequency of FRC used to build up the network. Results are shown either assuming a dense (blue) or a sparse (red) FRC network. In **D**, we calculate the centrality 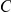 averaged over all cells (solid) or only FRC (dashed) dependent on the frequency of FRC used to build up the network. In (**C**,**D**), for each of the simulated values of 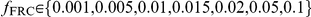 (dots) the average is taken over 100 different simulated networks. In **E** and **F**, we show the turning angle distribution of motile cells given either a dense (**E**) or sparse (**F**) network, respectively. The distribution is calculated over 500 different simulations for each of the different network densities considered.

To analyze the influence of different FRC network structures on the contact probability of two cells, we simulated FRC networks using different frequencies of FRC, 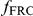, in a 3D cube consisting of 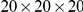 cells (see Figure 1A, B for a sketch of the model). For each value of 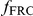 analyzed, we consider two different categories of networks: (i) a *dense* network, in which each FRC has contact to at least two other FRC, and (ii) a *sparse* network in which no condition on the connectivity of FRC is made. While the first situation corresponds to an intact network, the latter one represents a disrupted and impaired network as observed in different persistent infections. Before examining the contact probability of two cells on these networks, we will analyze the different network structures and their influence on cell motility with regard to experimental observations.

### In silico FRC networks and cell motility reflect experimental observations

In Figure 1C, we show the average fraction of the simulated space which would be surveyed by a cell crawling along all filaments of the FRC network. In a dense as well as in a sparse network, we observe an exponential increase in this value for increasing densities 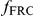. If 10% of the space is occupied by FRC, the whole simulated volume can be surveyed, independent of a dense or sparse network structure. This increasing coverage is associated with a reduced centrality value of a single FRC inside the FRC network (Figure 1D). The centrality, 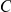, quantifies how likely a node in the network is reached by a cell which performs a random walk along the network (see *Materials & Methods* for a detailed description of the calculation) [27], [28]. In contrast, the average centrality of a random node in the cube is not affected by varying levels of 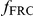. The average distance between two intersections of FRC filaments in our simulations is around 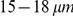 similar to experimental observations [15] (see *Supporting Information (SI)* Figure S2). Mueller et al. [21] used a tracer molecule to label the conduit system inside the white pulp in the spleen of mice. This basically represents the filaments of the FRC-network as they surround this system [24]. Image analysis revealed that roughly 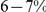 of the white pulp in the spleen of naïve mice is occupied by FRC and their filaments [21]. In our simulations, this would correspond to a dense FRC network constructed by 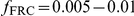, counting the frequency of edges representing FRC-filaments. The same parameterization for 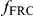, but in a sparse network, would occupy 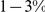 of the simulated space, comparable to the amount of FRC components observed in mice persistently infected with the LCMV Clone-13 strain [21].

In addition, we analyzed how different FRC networks affect the motility of moving cells. For the start, we assume that the FRC network only provides directional guidance for the movement of the cells and does not interfere with cell velocity. Simulated cells perform a random walk in the long term, as seen from the mean displacement of those cells (see *SI* Figure S3) and comparable to experimental observations [6]. In Figure 1E–F, the turning angle distribution of moving cells calculated over 500 independent simulation runs are shown, either for a dense or sparse network structure given different levels of 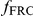. Each run followed 400 simulated time steps which corresponds to 280 min in real time. The rough pattern of the turning angle distribution, especially in a dense network situation with 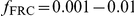, corresponds to the distribution observed experimentally for naïve T cells in a lymph node [7], [29]. Simulated T cells prefer small turning angles as determined by the way a new moving direction is chosen (see corresponding paragraph in *Materials & Methods*). There is nearly no change in the distribution of the turning angle between a situation without FRC network (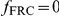) and when a FRC network is covering the whole simulated space (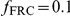, compare Figure 1C). These observations are robust against changes in the average length of filaments between FRC, 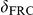.

### Disrupted network structure impairs contact probability of cells

**Figure 2:**
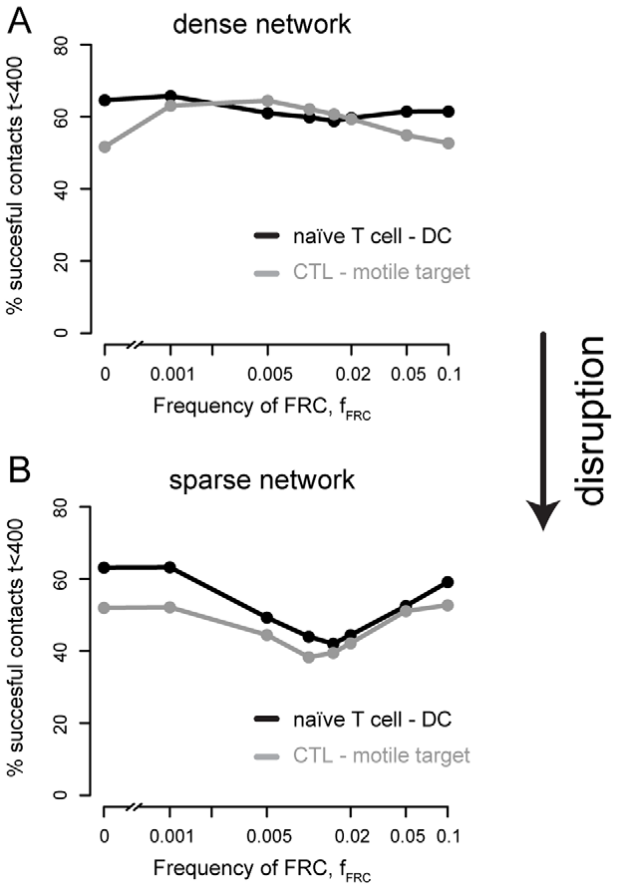
Contacts between cell pairs. **A** Frequency of successfully established contacts between a static dendritic cell and a naïve T cell and between a CTL and a moving target cell in case of a normal FRC network structure. Frequencies were determined for 5000 simulation runs followed over 400 timesteps each (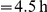 in real time). **B** shows the frequency of successfully established contacts in case of a disrupted FRC network structure. Please note, that the x-axis is shown on a logarithmic scale for graphical clarity.

In a first series of simulations, we investigate how a network structure affects the contact probability of two cells distinguishing between two different scenarios: (i) a naïve T cell interacting with a static dendritic cell with extracting dendrites, and (ii) a cytotoxic T cell hunting for a moving target. We determined the number of successfully established contacts for different FRC network scenarios after a maximum of 400 time steps. With one simulated time step corresponding to 0.7 min in real time, we are determining the contact rate of cells 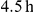 after they enter the lymphoid tissue. This is at the lower boundary of estimates for the dwell time of T cells in lymphoid organs, with these estimates varying between 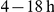 [30], [31]. We examined the search of a naïve T cell or CTL in the light of a dense or sparse underlying FRC network. If no FRC network is present, a contact between a naïve T cell and its corresponding static dendritic cell is established in around 64% of the cases after 400 simulated time steps (Figure 2). The success rate stays constant on this level (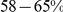) for different frequencies of FRC if these cells form a dense network structure (Figure 2A). In the scenario where a CTL hunts for a moving target cell, the probability of the hunting cell to find its target within 400 time steps equals 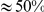 without any FRC network present. The contact probability slightly increases for increasing numbers of FRC, reaching a maximum value of 64% for 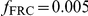 (Figure 2A). With 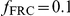, defining a FRC network covering the whole simulated compartment, the probability of a cell to find its target equals a scenario without any FRC network (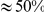).

If the network structure is disrupted and connecting filaments between FRC are lost, the contact probability of a cell with a static or motile target is reduced (Figure 2B). In the scenario with a naïve T cell interacting with a static DC, the success rate reaches a local minimum in 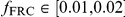. There, only 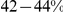 of the T cells will find their target during the simulated time period. A similar observation is made for the case of a CTL hunting for a motile target. For 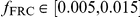, the success rate is only two third of the one observed in a dense network scenario.

**Figure 3:**
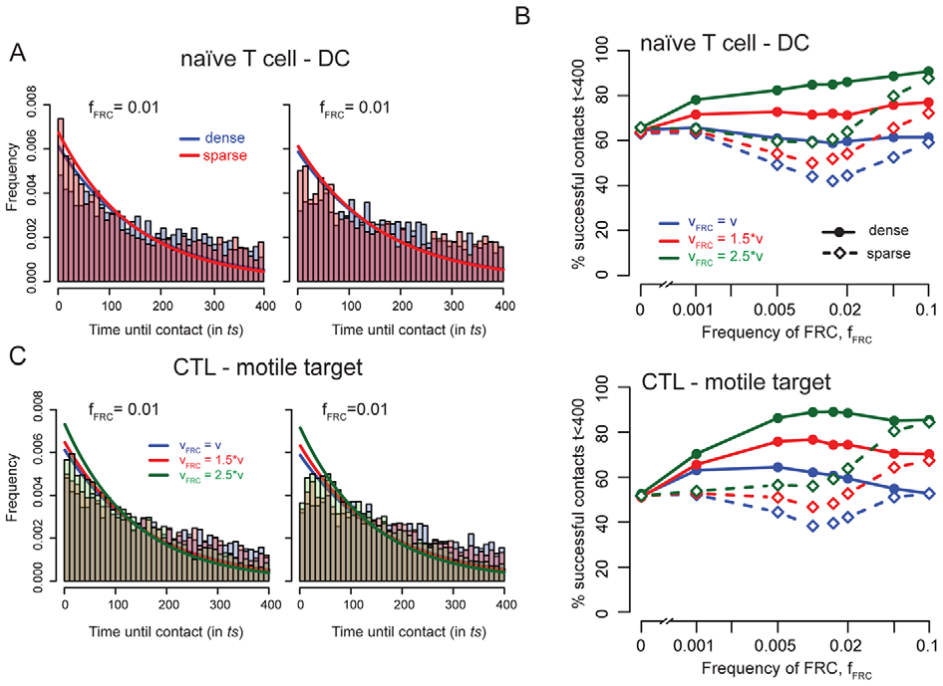
Time until contact and influence of velocity. In **A** we show the histograms for the time until a contact is made among all successful contacts either in a scenario with a static or moving target and separated for a dense (blue) or sparse (red) network scenario with 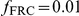. Solid lines represent the fit of an exponential distribution to these data (values for the rate constants and corresponding 95% confidence intervals are given in Table 1). Histograms represent several thousands successfully established contacts. In **B**, we examine the percentage of successfully established contacts in FRC networks that influence velocity. Results are shown for a static or moving target given a dense (solid line) or sparse (dashed line) network structure. For each value of 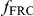 considered, the frequency of successfully established contacts is calculated over 5000 independent simulation runs, each followed over maximally 400 time steps (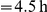 in real time). Panel **C** shows the histograms for the time until a contact is established among all successful contacts. Results are shown for a static (*left*) or moving (*right*) target cell in a dense network scenario with 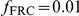. Solid lines represent the fit of an exponential distribution to these data (see also Table 1). Histograms are build on several thousands successfully established contacts.

We additionally examined, if the impaired contact probability of two cells is also reflected in a longer “hunting time” of the cells among those which succesfully established a contact to another cell. Figure 3A shows the histograms of the time until a contact is made among all successful contacts, either for a dense or sparse FRC network which is build up by a frequency of 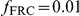 FRC. We fitted an exponential distribution function, which best describes such waiting time distributions, to the simulated data using a maximum likelihood approach. Our data indicate that the number of sufficiently established contacts follows an exponential distribution over time. The rate constants 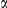 of the exponential distribution do slightly differ between the intact and impaired network (*naïve T cell - DC*: 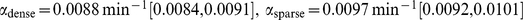/*CTL - motile target*: 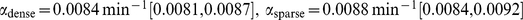). Numbers in brackets represent 95% confidence intervals of the estimates (see also Table 1). The results with 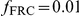 do not qualitatively change across the range of 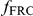 that we considered with 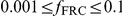. Furthermore, we examined if the time the naïve T cell or CTL needs to initially reach the FRC network affects the successful establishment of a contact to a DC or motile target cell, respectively. In neither of the scenarios analyzed did we find an influence of this time, or the initial distance of the two interacting cells at the beginning, on the establishment of a contact (Figure S5).

**Table 1:** Time until contact.

velocity increase	dense network	sparse network
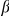	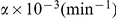	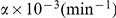
***static target*** **:**		
1	8.79 [8.45,9.1]	9.69 [9.27,10.09]
1.5	9.3 [8.98,9.6]	9.3 [8.92,9.66]
2.5	10.51 [10.2,10.84]	9.3 [8.95,9.64]
***moving target*** **:**		
1	8.44 [8.12,8.73]	8.8 [8.4,9.19]
1.5	9.09 [8.78,9.37]	8.8 [8.44,9.16]
2.5	10.29 [9.97,10.59]	8.73 [8.4,9.04]

Estimates for the rate constant 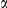 of an exponential distribution fitted to the data determining the time until a contact was made if a successful contact could have been established in 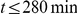 (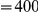 simulated timesteps). Numbers in brackets represent 95% confidence intervals.

### FRC networks affecting cell velocity increase contact probability of cells

So far we assumed that the FRC network only affects the moving direction of the cells. Several experiments showed that chemokines, such as the CCR7-ligands CCL19 and CCL21, are released by FRC and directly influence the motility of T cells [17], [18]. Worbs et al. [17] could show that these CCR7-ligands increased the median cell velocity by a factor of 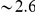. We incorporated this aspect into our simulations by increasing the velocity of cells which are connected to the FRC network. As long as a cell crawls along the simulated fibroblastic reticular fibres, the basic velocity 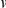 is increased by a factor 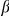, 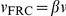, allowing a cell to perform more moves/swaps per time step. Results for 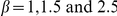 are shown in Figure 3B. With one simulated time step corresponding to 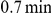 in real time, this leads to average cell velocities in the range of 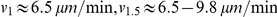 and 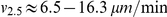 (compare to [17]). With 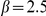 and 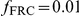, corresponding to cell velocities observed *in vivo*, the motility coefficient of simulated cells shows reasonable values (see *SI* Figure S3). In a dense network scenario, the contact probability of two cells is slightly increased for increasing cell velocities (compare Figure 3B). When a CTL is hunting for a moving target and a dense FRC network is provided (Figure 3B, *lower panel*), the maximal probability to successfully find their counterpart is observed for 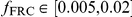, irrespectively of the network affecting cell velocity. If the network structure is disrupted, the probability of a cell to find its target is maximally reduced in this parameterization. This latter observation is also valid in the case of a naïve T cell - DC scenario.

The increase of the cell velocity by a factor of 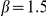 due to the FRC network does not significantly affect the time until a contact between two cells is made (Figure 3C, Table 1) (*naïve T cell - DC*: 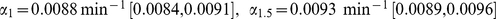/*CTL - motile target*: 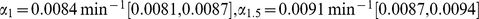). If 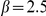, there is a slight tendency for cells to find their counterpart earlier than for lower values of 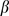 (*naïve T cell - DC*: 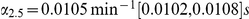/*CTL - motile target*: 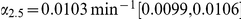). The same analyses were performed using a larger simulated volume of 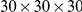 cells, which led to the same results (see *SI* Figure S4).

## Discussion

Several studies showed that disruption of the FRC network correlates with a disturbed organization of the lymphoid tissue and leads to the loss of control of an ongoing infection. The network of fibroblastic reticular cells inside secondary lymphoid organs affects cell motility, and is assumed to facilitate the interaction between different cells in the LN or spleen [11], [21], [24], [32], [33]. However, a “road system” for cells, as e.g. represented by the FRC network, provides guidance, but also constraints. A sparse road system is difficult to reach, but once a cell moves on the roads it will easily find its target. A dense road system, on the other hand, is reached fast, but it may not be any easier to find a target than in free space because there are too many routes available. This trade-off between guidance and constraints gives rise to an optimal network density.

The quantitative relationship between cell interaction and FRC network cannot be investigated experimentally at present because it requires the manipulation of the FRC network structure. Therefore, we investigated this relationship and the trade-off as it is described above using a simulation model for the movement of T cells in a lymphatic tissue. We extended a 3D cellular automaton of a lymphoid region inside the spleen which we developed previously [26]. We specified the FRC network structure and rules which define the movement of simulated cells along an FRC network. The resulting network and cell motility characteristics is consistent with experimental observations [6], [15], [29].

To simulate biologically plausible cell behaviour in our model, the moving direction of a cell depends on two components. The first component is the intrinsic movement behaviour of a cell. As a change in moving direction requires a costly restructuring of the actin-cytoskeleton [34], cells prefer small turning angles. The second component influencing the moving direction of cells in our model is the FRC network. The fraction of time a simulated cell follows the filaments of the FRC network is around 80% and varies dependent on the density of the FRC network. While not consistently moving along the network, this fraction is always higher as you would expect by just random movement without influence of the FRC network on the movement direction. The turning angle distribution is therefore influenced by the FRC network and, hence, the frequency of filaments in the simulated volume (see Figure 1E and F). However, the intrinsic movement behaviour prevents cells to solely follow this network as this might require cell turns that are overruled by the preference for small turning angles. This reduces the differences between the turning angle distributions for different kind of networks. We neglected to model cell movement without the intrinsic movement behaviour solely following the FRC network in order to simulate more biologically plausible cell behaviour.

Our analysis suggests that the fibroblastic reticular network as a guidance system has only a small effect on the probability of cell encounters. However, if the FRC network is disrupted by losing some of the filaments the probability of two cells to find each other is reduced. This is observed for static targets, such as dendritic cells, and moving targets alike. It has been previously shown that the absence or alteration of the FRC network inside LNs and the spleen also impairs the motility of moving lymphocytes [16], [18], [32]. Therefore, we investigated how an increase of cell velocity along the filaments of the FRC network affects the contact probability. Assuming this additional property of the FRC network, the influence of varying FRC network densities on the probability of a cell to find either a moving or static target slightly increases. However, this did not affect the relative effect of a disruption of the FRC network on the contact probability between cells.

In general, we found that the presence of FRC network increases the contact probability between two moving cells by approximately 20–25%, or even up to 40% if the FRC network also contributes to cell velocity. In contrast, the probability of a moving T cell to encounter a static dendritic cell is unaffected by the FRC network. This is probably due to the fact that in the latter situation the protruding dendrites of the dendritic cell survey a large fraction of the simulated volume. Even considering a larger simulated volume (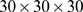 cells) led to the same results (see *SI* Figure S4). However, we have to emphasize that our simulations represent a more challenging environment for the activation of a specific naïve T cell than in biological plausible conditions. The T cell - DC ratio in our simulations (1∶1) is even lower than observed in two-photon microscopy experiments which study T cell and DC interaction. In these experiments, usually 1–2% of all T cells in a lymph node are fluorescently labelled and appropriate DC make up about 0.5% of all cells (roughly 10–30% of all DC). Based on these estimates, roughly 2–5% of the cells in a LN are DC and, for simplicity, we assume that the rest of the cells are T cells as we are modeling the T cell zone of a secondary lymphoid organ [35]. The total volume of a DC is assumed to be around 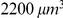 [8], [35]. Under the assumption that 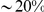 of the simulated space is occupied by reticular network or represents free space, we would have to model 2–15 DC and 60–120 specific T cells in a cube of 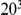 nodes (6–50 DC vs. 200–400 T cells for 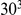).

We study the first contact between two cells given a fixed ratio of the two cell populations considered. In order to leave space unconfined, we use periodic boundary conditions in our cellular automaton. We checked that the periodic boundary conditions do not affect our results. While during a simulation on average around 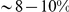 of the moves of a cell “pass” the periodic boundary, we found no difference in this value, or its variation, among all the different scenarios considered (static/moving target, dense-sparse FRC networks, different FRC-frequencies). There is also no difference between the runs which ended in a contact between the cells or those which did not after a period of 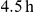. Therefore, the comparisons we show are not biased in the one way or the other by the periodic boundary conditions of the simulation system.

Quantification of the first-passage time, the time a random walker needs to reach a certain target point, plays an important role in different kinds of target search processes [36], [37] or the dynamics for the spread of diseases [38]. Applying previously developed mathematical theory calculating first-passage times [39], [40] to the scenario described here can be used to corroborate the simuation results. However, such a theoretical approximation has to be carefully compared to the complex migration characteristics and dynamics, which was beyond the scope of this study, and will be subject to future work.

While in total the observed increase in cell contact probability due to a dense FRC network is only small, which is in line with recent simulation results confining the movement of cells to the network [41], it remains to be investigated how an increase of 20% to 30% in the efficacy of cell encounter affects the susceptibility to infection, the morbidity due to infection, and finally the fitness of the host. We found that the FRC network density observed in the spleen of naïve mice [21] corresponds to a density that is maximally vulnerable to disruption. However, the evolutionary pressure evoked by this disadvantage might be too low to favour the development of alternative network structures. Furthermore, if the FRC network does not enhance the contact probability of an individual naïve T cell to a DC substantially as shown in the simulations, the question remains if the FRC network provides additional factors for the proper function of the immune response which are not captured in the simulations so far. In the following, we propose several hypotheses and possible experimental approaches to test them.

One hypothesis porposes the FRC-network to represent something like a crowd control system to improve DC scanning rates. The average distance between two filaments of the FRC-network is observed to resemble the average diamter of a T cell [11], [12], [15]. This could force T cells to move in streams through the lymph node. While the time of an individual T cell to reach a certain DC might not be increased by this, it could allow DC, which are attached to fibroblastic reticular cells, to scan more different T cells per minute. Without the network, cells might clump around DC, limiting access to the dendritic cell. This hypothesis is corroborated by several experimental and simulation studies [8], [26], [42], which proposed that the contact duration, i.e. the time a DC needs to scan a T cell (attach - detach), is more important for efficacy than the time it actually takes to find a corresponding cell. Previous simulation studies have already observed the occurence of T cell streams along the filaments of the FRC network [35]. The modeling frameworks used in this study might be useful to examine this hypothesis theoretically. One possibility to investigate experimentally the hypothesis that the FRC network works as a crowd control system could be to perfom two-photon imaging analysis of T cell - DC interactions in lymph nodes of naïve mice and those that show an impaired FRC network structure, as e.g. evoked by persistent infections in mice [21]. Accounting for confounding factors, such as the presence of an infection in the persistenly infected mice, one could check if the number of individual T cells scanned by one DC differs between the two situations. However, one has to ensure that no other factors of the persitstent infection impair the comparison.

Besides interferring with the motility of T cells, the FRC network could also facillitate cell encounters by trapping immune cells inside the lymphoid organs. Estimates for the dwell time of a T cell in a lymphoid organ vary around 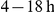, for B cells between 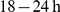 [30], [31]. By presenting “homing” molecules, such as CCL19 and CCL21, the FRC might contribute to the length of the time a lymphocyte will stay in the spleen or LN, respectively. Without the network, the transit time of a T cell through the spleen and thereby its chance to find its cognate antigen could be reduced. This might even increase the damage induced by network impairment, as we found that, for biologically relevant cell velocities, cells find their counterpart earlier in a dense than in a sparse network (see Table 1, 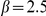). Transfer of labelled T cells into the spleen of naïve mice and those showing an impaired FRC network can be used to estimate the dwell time of T cells in the spleen of the two different animal populations, and to detect differences.

Recently, a new experimental approach was presented to construct FRC networks *in vitro* on a macroporous polyurethane scaffold [43]. Constructing FRC networks on this scaffold with different doses of FRC clones and using appropriate imaging techniques might show if varying FRC network densities affect the number of contacts between cells. Varying the FRC network density, and analyzing cell motility and interactions *in vitro* could allow us to quantify the effect of FRC network disruption on immune cell dynamics in more detail.

## Materials and Methods

### The cellular automaton

The interaction of naïve T cells/CTL with dendritic cells (DC)/target cells, as well as with the FRC network is simulated on two separate but interacting three-dimensional lattices of nodes and edges, unlike our previous model [26]. Each node denotes the body of a cell and has 26 direct neighbours. Edges define paths on which FRC fibres can grow or cells are able to move. We define periodic boundary conditions in which a cell, or a dendrite or fibre of a DC or a FRC, respectively leaving the simulated space on the one side of the lattice reappears on the opposite side. The cellular automata were implemented in the C++ programming language.

#### Construction of the FRC network

On one 3D grid of nodes and edges, we grow the reticular network which is spanned by FRCs and their filaments. This grid interacts with the separate grid containing the T cells and target cells by influencing their movement and motility (see below and Figure 1A). The structure of the reticular network is determined by two quantities: (i) the frequency of FRC spanning the reticular network, which means the frequency of nodes in the grid occupied by a FRC, 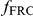, and (ii) the minimum number of other FRC one FRC has to be connected to, 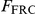. The FRC network is then constructed as follows: One FRC is randomly placed on a node in the lattice. Out of this node, a fibre grows for a predefined distance 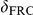 along the edges in the lattice, choosing a new direction randomly at each intersection it reaches. A new FRC will be placed on the node at the tip of this fibre. The new FRC serves as a root for the next fibre. This process is repeated until a number of 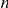 FRC are seeded (default 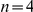). Then a new FRC root will be randomly placed on a node in the lattice and the whole process continues until the predefined frequency of FRC, 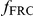, is reached (see also *Supporting Information*, Figure S1A). Additionally to the reticular fibres, which are built during this growing process, FRC launch shorter filaments which do not need to have a connection to other FRC. The default number of steps for the growing filament between two FRC is set to 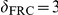. This would correspond to a distance of 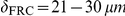 between FRCs, if we assume that the edgelength is determined by the diameter of a T cell (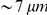) occupying one node [15]. We manipulate the actual FRC network structure by varying the frequency of FRC in the simulated space, 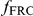. Additionally, in order to construct a *dense* network, we define the minimum number of connections ( = fibres) one FRC has to have to other FRC to be 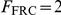. If the actual number of connections of a FRC is below 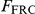, a new fiber to a randomly chosen FRC will be built. For a *sparse* network, there is no minimum number of direct paths defined. Once the FRC network structure is built, the network is kept constant during the simulation run.

#### The lattice for cell movement

In a second lattice, we consider the different interacting cell populations such as naïve T cells and DC or CTL and infected target cells, respectively dependent on the scenario we are looking at. This lattice superimposes the lattice which contains the reticular network (see Figure 1A). Naïve T cells, CTL as well as motile target cells occupy one node in the simulated lattice, having an average diameter of 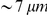 and an average volume of 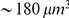 [12]. They can be positioned anywhere in the lattice upon initialization. Dendritic cells consist of a cell body as well as dendrites, which comprise roughly 2/3 of the total cell volume that is assumed to be around 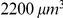. This would correspond to a diameter of a DC of 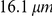 given a spherical shape [8], [35]. The dendrites could be protruded into every direction determined by the edges attached to the cell body. These dendrites are simulated to continuously extend and retract from the cell body while the total volume of the DC is kept constant. DC preferentially adhere to FRC [19], [20], [24]. Therefore, they are initialized on a position which is in the direct neighbourhood or on the position of a FRC itself compared to the lattice which comprises the FRC network structure. After having initialized the pair of specific cells (naïve T cell-DC/CTL-infected target), the rest of the lattice is filled with unspecific lymphocytes. Some of the nodes are left unoccupied and correspond to free space. As lymph nodes and the spleen are densely packed organs, approximately 2.5% of the nodes denote free space.

### Simulating cell movement

Each cell that is capable to move, such as naïve T cells or CTL, possesses a certain moving direction 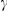. The moving direction 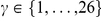, pointing to one of the 26 neighbouring cells, can change during a simulation run as described below. We distinguish between two types of movement as done before [26]: Either cells move into free space or they swap their place with a neighbouring cell. Thereby, a naïve T cells or CTL swaps its place with an unspecific splenocyte irrespectively of the moving direction of this cell. The movement of cells is constrained by the underlying FRC network. Each edge in the lattice of the FRC network is weighted according to the connection and distance to an FRC. This weight 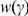, 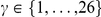, represents the amount of chemokines released and defines the level of attraction of a moving cell into this direction. The weight is determined in such a way that the attraction to a FRC is higher than the movement away from it. Edges without FRC filaments receive a weight 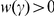, roughly 100 times lower than those with FRC elements. This assumption ensures, that cells preferentially crawl along the FRC network as observed experimentally. Some edges close to FRC are assigned a weight of 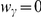 which accounts for paths that are blocked by spatial obstacles [26], [35]. The probability of an edge connected to a FRC to have a weight of 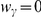 depends on the frequency of filaments connected to this FRC in accordance to the biological situation. The more filaments are connected, the more likely it is that some pathways into other directions are blocked by those filaments.The number of pathways blocked is sampled from a uniform distribution over 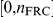, where 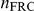 denotes the number of different filaments connected to this FRC. However, we have to emphasize that, although edges are blocked, each node in the lattice can be reached, e.g. from a different side. Dependent on the frequency of FRC and the density of the FRC-network, on average 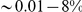 of all possible moving directions are blocked.

As cell movement requires a complex restructuring of the actin-cytoskeleton [34], cells prefer small turning angles 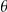. Therefore, a second weight 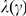 is assigned to every moving direction 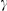 which incorporates the turning angle 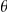 between 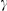 and the previous moving direction 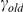. The calculation of 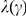 is similar to the direction assignment performed in [29] and comparable to the assignment done before [26]. The weight into direction 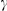 is determined by the distance to the surface of a composite ellipsoid which includes 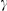 and 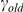 and is defined by
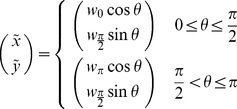
(1)The parameters 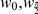 and 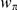 are predetermined and fixed for all simulations. The default parameterization is defined by 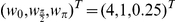 (see *SI* Figure S1C for an illustration of the weight assignment). With this default parameterization we ensure a preference of the moving cell for small turning angles. Varying the actual values, but still keeping a preference for small turning angles, did not change our general results.

Before a cell makes a move, the direction 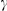 of the cell is determined according to both these weights. The probability 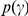 to move into direction 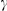 is calculated by:
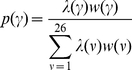
(2)The actual new moving direction of the cell is then sampled from this multinomial distribution.

### Contact of cells

A simulated CTL is assumed to be in contact to a target cell if these cells are on neighbouring nodes [26]. As the average distance between two nodes is assumed to be 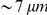, which corresponds to the average diameter of a T cell, the membranes of cells on neighbouring nodes would attach to each other and, therefore, a contact is counted. A simulated naïve T cell is assumed to have established a contact with a static dendritic cell either if the naïve T cell is located on a node directly connected to the node were the core of the DC is situated, or if the position of the naïve T cell is reached by a dendrite of the DC. Thereby, a cell is assumed to be reached by the dendrite if the dendrite reaches or covers the node the T cell is occupying. If the position of the naïve T cell is only insufficiently reached by one of the continuously varying dendrites, we calculate the distance of the tip of the dendrite to the node the T cell is occupying, defined by the parameter 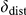, with 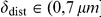. The probability that a contact is established in this situation is then sampled from a binomial distribution with 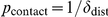. This scenario refers to the fact that the membrane of the T cell might as well be in connection to the dendrite as our model does not cover the actual flexible shape of a T cell.

### Simulation

Simulation runs are performed as follows: First, the cellular automaton with the FRC network is constructed according to the assigned frequency of FRC, 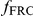. Second, the cellular automaton containing the cells which actually move along the FRC network is initialized. Thereby, we examine either a situation with a motile naïve T cell and a static DC (in the following referred to as *static target*) or with two motile cells (*moving target*). The second scenario corresponds to a CTL hunting for an infected target cell. During one simulated time step, each motile cell will sample a moving direction 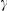 as described above and perform a move into this direction if possible. Furthermore, each cell is able to move into free space (compare to [26]). While motile cells move, a DC will extend and retract its dendrites. After each time step, it is checked if the specific cell pair has made a contact. A simulation will run for 400 time steps or until a contact is established. A time step corresponds to 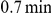 in real time, hence, 400 time steps correspond to 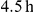 in real time. Estimates for the dwell time of T cells in lymphoid organs such as lymph nodes or the spleen vary around 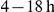 [30], [31]. We chose the lower boundary of the estimates of 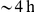 for our analyses to determine the success rate for the fastest passage time of T cells. For each randomly constructed FRC network, 50 independent runs of cell pairs are performed.

### Characterization of the fibroblastic reticular network

In our simulation environment, we have to distinguish between two types of networks on which the cells can move. The lattice-network comprises all nodes and edges in the 3D cellular automaton cube. This network specifies all possible moving directions of a cell, 26 possible edges at each node. The actual FRC network is a subset of those edges and only consists of the FRC and the connecting edges which represent the filaments of the FRC network. These ones include the weights of the FRC network. The FRC network can be characterized by several parameters. First of all, as described above, it is defined by the frequency of FRC spanning up the network, 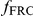, as well as the connectivity of the FRC determined by 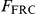. Additionally, we define different parameters to describe the structure of the FRC network: (i) the centrality of a node or a FRC in the total lattice-network or FRC-network, respectively, 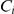 and (ii) the “area surveyed by the FRC network”. The centrality 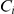 of each node 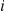 is a quantity used in general network analysis. This measurement quantifies, how likely a node in the network is reached by a cell which is randomly crawling along the lattice [27], [28]. To calculate 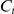, we determine the degree 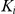 of each node which determines the sum over all weights of the edges connected to this node, 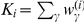. The centrality 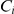 for node 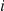 is then calculated by the following formula [27]:
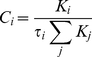
(3)Hereby, the sum over 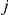 is taken over all neighbouring nodes of node 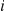. For simplicity, we define 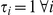. We calculate the centrality 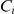 among all nodes in the 3D lattice as well as only for the fibroblastic reticular cells in the FRC network (see Figure 1D). The second parameter, the “area surveyed by the FRC network”, (see Figure 1B) is defined by the volume a T cell is able to scan while crawling along all edges of the FRC network (see *Supporting Information*, Figure S1B).

## Supporting Information

Figure S1
**The FRC network and cell moving direction.** **A** A sketch for the construction of the FRC network in the simulation in 2D. An FRC (yellow circle) is seeded on a random node in the lattice from which a fibre will grow for a total of 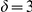 steps (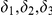), where each step comprises an edge to a neighbouring node. At the ending node, a new FRC is seeded and the process is repeated. To construct a dense network, additional connections between FRC are constructed (dashed line) to ensure that each FRC has contact to at least two other FRC. **B** The area surveyed by the FRC network (grey shaded area) is given by the nodes of the grid which a T cell (blue circle) would reach while crawling along the network. **C** Sketch for the assignment of weights 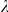 for new possible moving directions 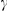 dependent on the turning angle 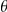. The old moving direction 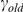 is shown in red. The weights 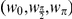 determine the spanned surface area of the ellipse. The length of the blue arrow would define the weight 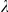 for a movement into this direction. The ellipse is shown in 2D, for 3D imagine that the ellipse rotates around the 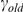-axis.(TIF)

Figure S2
**Average distance between intersections of FRC filaments.** For each value of 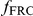, we show the median average distance between two intersections (nodes with at least three connected FRC filaments) determined over the average distance calculated for 100 different dense FRC networks. Arrows denote the maximal and minimal average distance seen in these 100 simulated FRC networks.(TIF)

Figure S3
**Mean displacement of moving cells.** **A** The mean displacement against the square root of time in a dense FRC network with 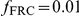 that affects cell motility and velocity. The motility coefficients 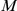 calculated as in [26] are 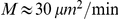 (blue), 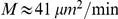 (red) and 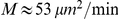 (green). In **B** we show the mean displacement against the square root of time for different dense FRC networks which increase cell velocity by a factor of 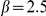 (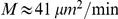 (red), 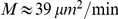 (blue), 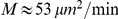 (green), 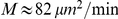 (orange)). Each curve is calculated with the mean displacement over 100 simulated cells followed over 400 time steps. Each time step corresponds to 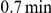 in real time.(TIF)

Figure S4
**Percentage of successfully established contacts.** Percentage of successfully established contacts with a FRC network that influences cell motility and velocity assuming a cubic space of 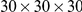 cells. Results are shown for a static (**A**–**C**) or moving target (**D**–**F**) given a dense (solid line) or sparse (dashed line) network structure. For each value of 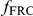 considered, the frequency of successfully established contacts is calculated over 5000 independent simulation runs, each followed over maximally 400 time steps. The shaded areas correspond to the average cell velocity calculated over all simulations either for the dense (light) or sparse (dark) network structure. Thereby, one simulated time step would correspond to 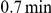 in real time.(TIF)

Figure S5
**Contact to the network.** Initial time a naïve T cell needs to reach the FRC-network (**A**), and initial distance at the start of the situation (**B**). Boxplots are shown separately for simulations which ended in the successful establishment of a contact to a DC after 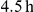 (*light grey*), and those which did not (*dark grey*), given either a dense or sparse network with 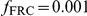 or 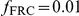. The time to reach the FRC-network, as well as the distance at the beginning, does not seem to have an influence on the successful establishment of a contact between a naïve T cell and a dendritic cell.(TIF)
