# Supplementary figures and images for: Influence of the Fibroblastic Reticular Network on Cell-Cell Interactions in Lymphoid Organs
Source: PLoS Comput Biol. 2012 Mar 22;8(3):e1002436. doi: 10.1371/journal.pcbi.1002436 (PMC3310707; doi:10.1371/journal.pcbi.1002436)

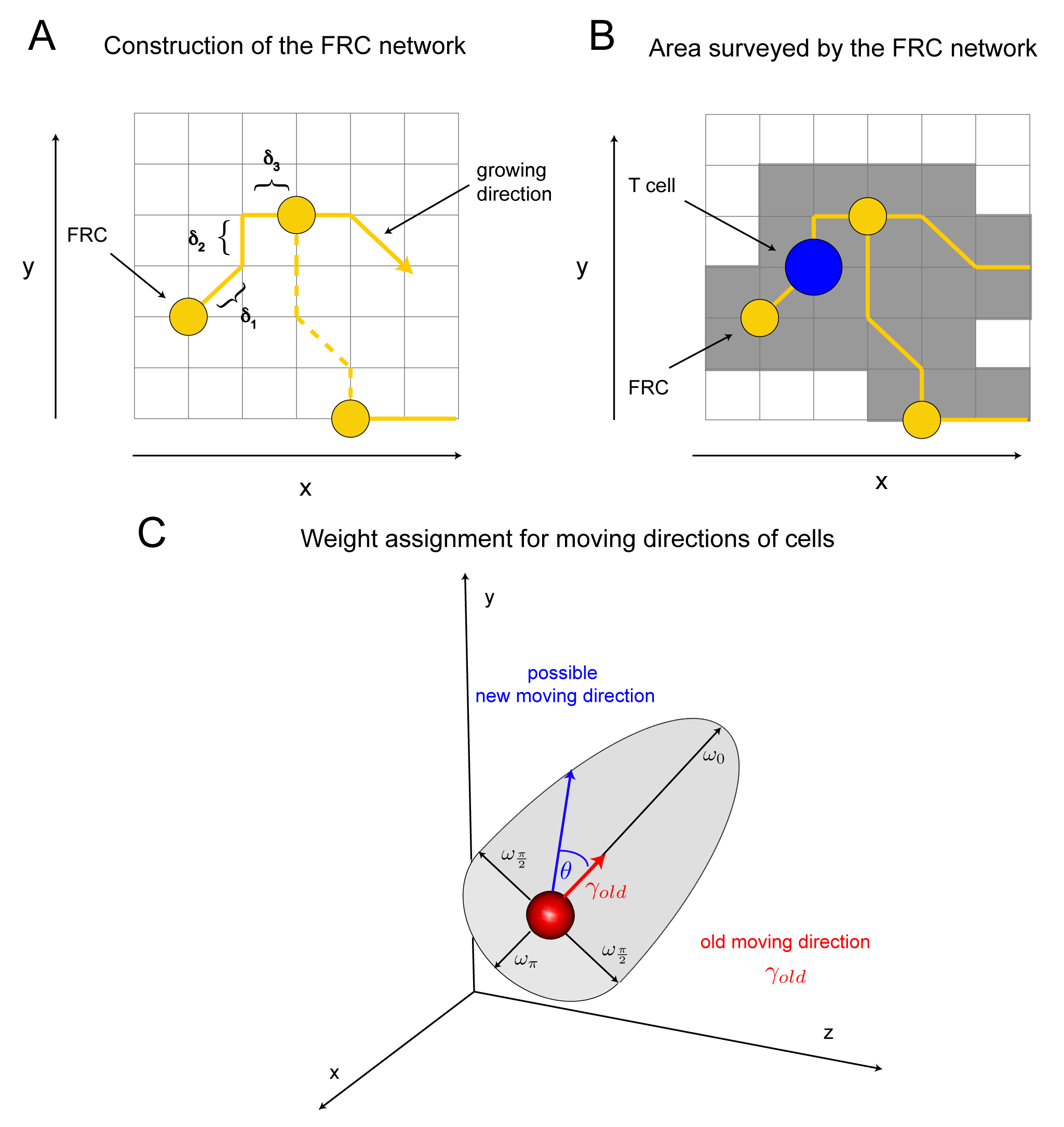

Supplement: Figure S1 — The FRC network and cell moving direction. A A sketch for the construction of the FRC network in the simulation in 2D. An FRC (yellow circle) is seeded on a random node in the lattice from which a fibre will grow for a total of steps (), where each step comprises an edge to a neighbouring node. At the ending node, a new FRC is seeded and the process is repeated. To construct a dense network, additional connections between FRC are constructed (dashed line) to ensure that each FRC has contact to at least two other FRC. B The area surveyed by the FRC network (grey shaded area) is given by the nodes of the grid which a T cell (blue circle) would reach while crawling along the network. C Sketch for the assignment of weights for new possible moving directions dependent on the turning angle . The old moving direction is shown in red. The weights determine the spanned surface area of the ellipse. The length of the blue arrow would define the weight for a movement into this direction. The ellipse is shown in 2D, for 3D imagine that the ellipse rotates around the -axis. (TIF) [file pcbi.1002436.s001.tif]

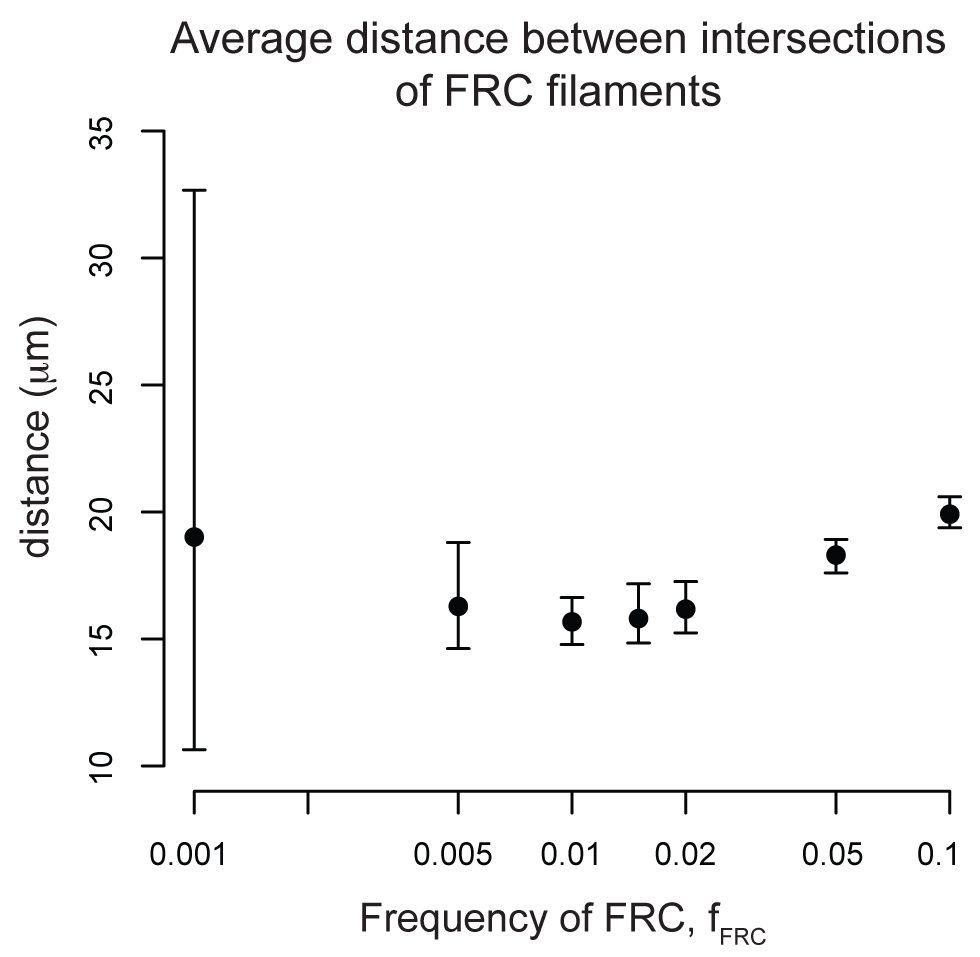

Supplement: Figure S2 — Average distance between intersections of FRC filaments. For each value of , we show the median average distance between two intersections (nodes with at least three connected FRC filaments) determined over the average distance calculated for 100 different dense FRC networks. Arrows denote the maximal and minimal average distance seen in these 100 simulated FRC networks. (TIF) [file pcbi.1002436.s002.tif]

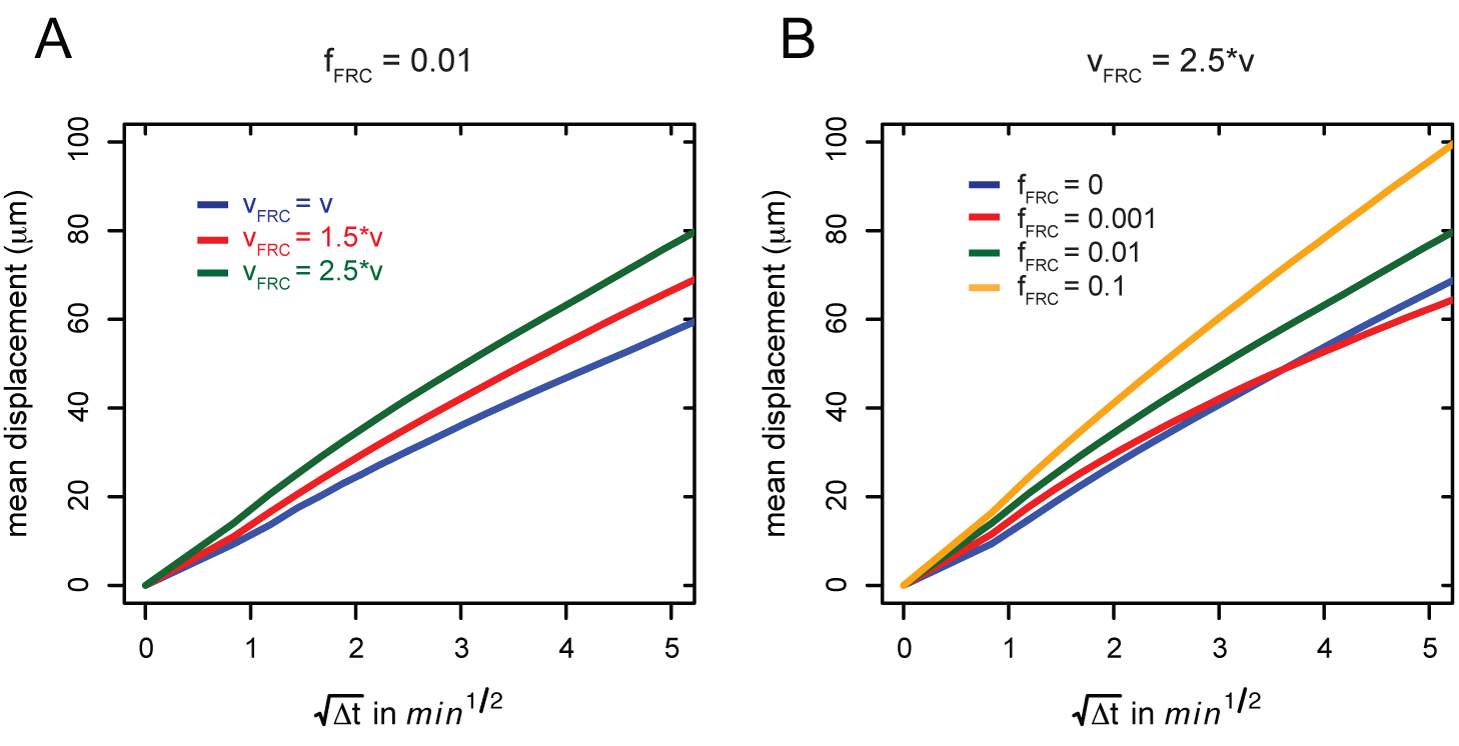

Supplement: Figure S3 — Mean displacement of moving cells. A The mean displacement against the square root of time in a dense FRC network with that affects cell motility and velocity. The motility coefficients calculated as in [26] are (blue), (red) and (green). In B we show the mean displacement against the square root of time for different dense FRC networks which increase cell velocity by a factor of ( (red), (blue), (green), (orange)). Each curve is calculated with the mean displacement over 100 simulated cells followed over 400 time steps. Each time step corresponds to in real time. (TIF) [file pcbi.1002436.s003.tif]

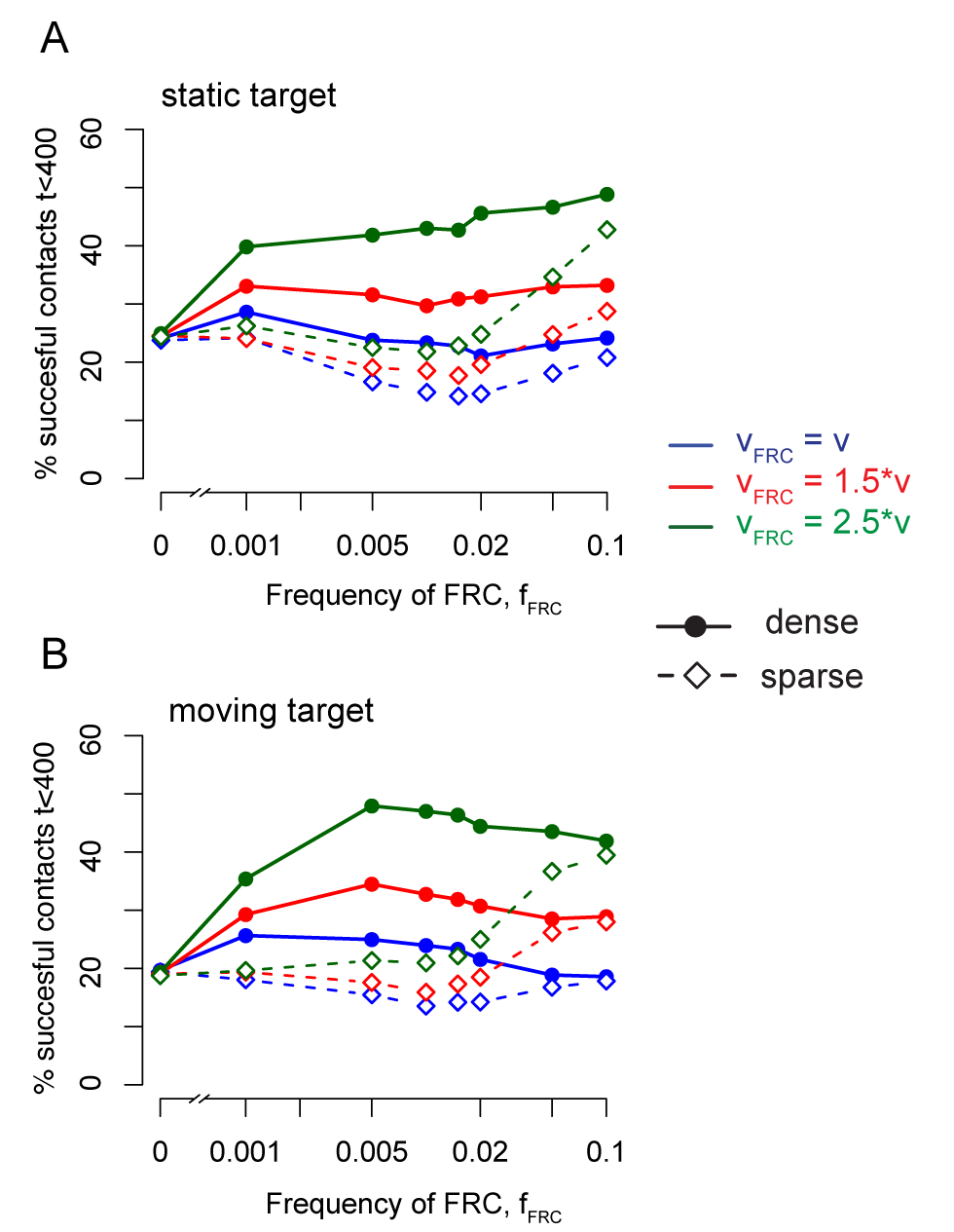

Supplement: Figure S4 — Percentage of successfully established contacts. Percentage of successfully established contacts with a FRC network that influences cell motility and velocity assuming a cubic space of cells. Results are shown for a static (A–C) or moving target (D–F) given a dense (solid line) or sparse (dashed line) network structure. For each value of considered, the frequency of successfully established contacts is calculated over 5000 independent simulation runs, each followed over maximally 400 time steps. The shaded areas correspond to the average cell velocity calculated over all simulations either for the dense (light) or sparse (dark) network structure. Thereby, one simulated time step would correspond to in real time. (TIF) [file pcbi.1002436.s004.tif]

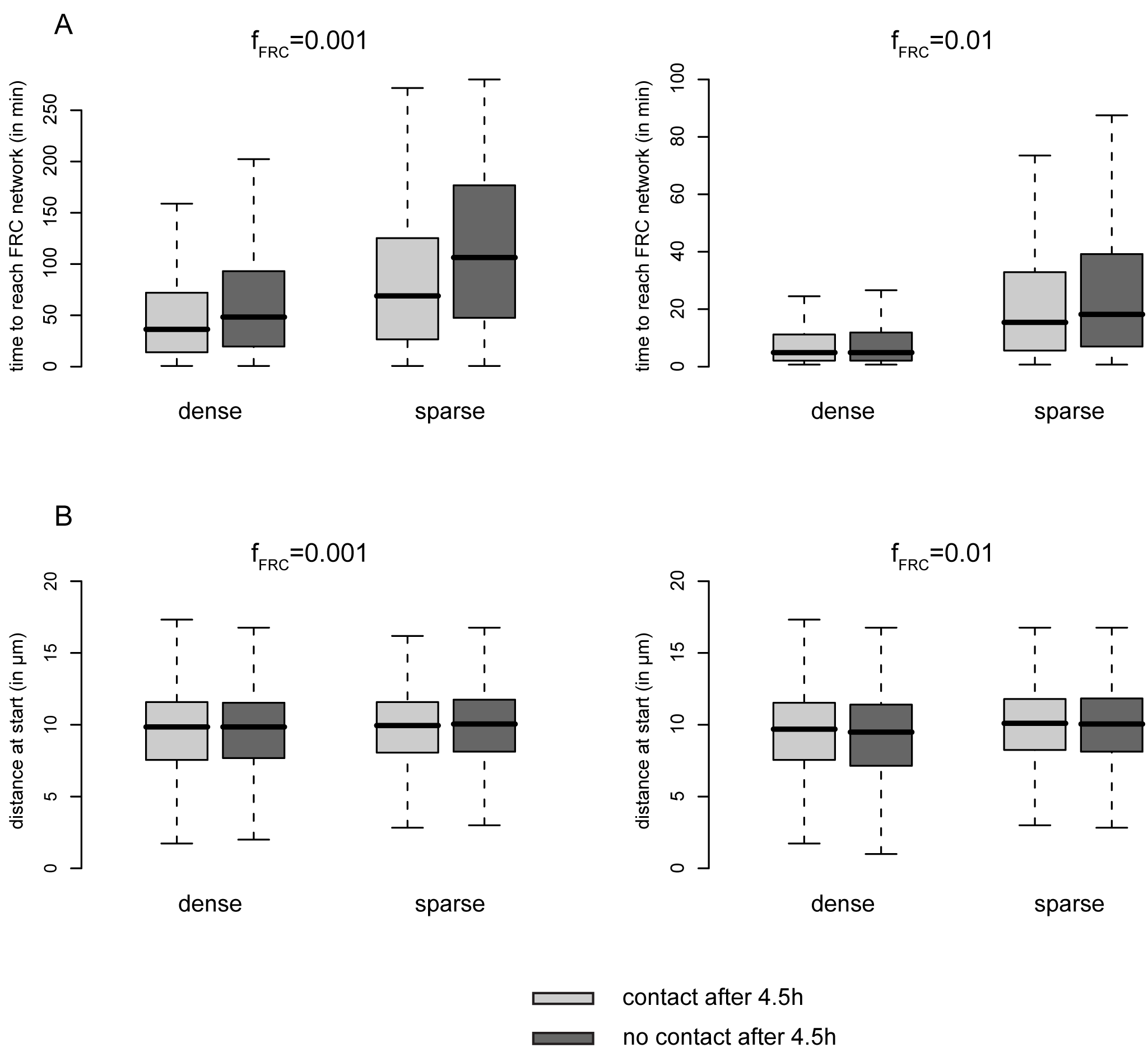

Supplement: Figure S5 — Contact to the network. Initial time a naïve T cell needs to reach the FRC-network (A), and initial distance at the start of the situation (B). Boxplots are shown separately for simulations which ended in the successful establishment of a contact to a DC after (light grey), and those which did not (dark grey), given either a dense or sparse network with or . The time to reach the FRC-network, as well as the distance at the beginning, does not seem to have an influence on the successful establishment of a contact between a naïve T cell and a dendritic cell. (TIF) [file pcbi.1002436.s005.tif]
